# Subacute Ruminal Acidosis in Zebu Cattle: Clinical and Behavioral Aspects

**DOI:** 10.3390/ani11010021

**Published:** 2020-12-24

**Authors:** Natalia Sato Minami, Rejane Santos Sousa, Francisco Leonardo Costa Oliveira, Mailson Rennan Borges Dias, Débora Aparecida Cassiano, Clara Satsuki Mori, Antonio Humberto Hamad Minervino, Enrico Lippi Ortolani

**Affiliations:** 1 Department of Clinical Science, College of Veterinary Medicine and Animal Science, University of São Paulo (FMVZ/USP), 05509-270 São Paulo, Brazil; minaminatalia9@gmail.com (N.S.M.); rejane.santossousa@gmail.com (R.S.S.); oliveiraflc@usp.br (F.L.C.O.); mailsonveterinario@gmail.com (M.R.B.D.); debora.apcassiano@gmail.com (D.A.C.); clarasat@usp.br (C.S.M.); 2 Laboratory of Animal Health, LARSANA, Federal University of Western Pará, UFOPA, 68040-255 Santarém, Brazil

**Keywords:** SARA, Nelore, feeding behavior, citrus pulp, clinical picture

## Abstract

**Simple Summary:**

Cattle that are fed high levels of concentrates may develop short-term rumen acidity that may occur frequently leading to necrosis of the rumen wall and reduced nutrient absorption, thereby decreasing animal productivity. This condition is known as subacute acidosis. Here, we evaluated an experimental model to induce such a condition in Nelore cattle, a Zebu breed widely used in Brazil, and assessed several clinical and feeding behavioral patterns of affected animals to better understand the disease pathogenesis and clinical outcomes. Subacute acidosis led to a reduction in food consumption and rumination time, and an increase was observed in the time spent in decubitus. Additionally, subacute acidosis caused different degrees of depression that was more pronounced with higher ruminal lactic acid concentrations.

**Abstract:**

We evaluated the clinical aspects and feeding behavior of cattle with subacute ruminal acidosis (SARA) caused by short-chain fatty acids (SCFAs). Ten healthy Nelore heifers were subjected to an adjusted SARA induction protocol using citrus pulp (CP). Clinical examinations were performed at baseline and at 3, 6, 9, 12, 15, 18, and 24 h intervals after induction, with ruminal fluid, blood, and feces sampling. The animals’ feeding behavior was evaluated on, before, and for 3 days after SARA by observing the animals every 5 min for 24 h. The dry matter intake (DMI) was recorded daily. The ruminal pH during SARA was always lower than baseline, with an acidotic duration of 547 ± 215 min, a minimum pH of 5.38 ± 0.16, and an average pH of 5.62 ± 0.1. SARA was mainly caused by SCFAs (maximum 118.4 ± 9.3 mmol/L), with the production of l-lactic acids (7.17 mmol/L) and d-lactic acids (0.56 mmol/L) 6 h after the experiment began. The DMI was reduced by 66% and 48% on days 1 and 2, respectively, and returned to normal levels on day 3. SARA caused a reduction in feed intake and rumination time, as well as an increase in the time spent in decubitus on days 1 and 2. These results were influenced by the ruminal pH, ruminal movement, and osmolarity. Furthermore, SARA caused different degrees of depression, which became more pronounced with higher ruminal lactic acid concentrations.

## 1. Introduction

Animals in confinement and semiconfinement systems are required to achieve greater productivity levels (e.g., higher weight gain and feed conversion rates). These production goals require increased use of high energy diets, which are rich in concentrates and favor productive efficiency. However, at the same time, such diets increase the frequency of the occurrence of digestive diseases, especially ruminal acidosis [1,2,3].

Acidosis occurs by the ruminal fermentation of soluble carbohydrates present in starchy grains, such as corn, sorghum, and citrus pulp (CP). There is an exacerbated production of short-chain fatty acids (SCFAs) that results in a mild case of ruminal acidosis, with few clinical manifestations in the affected animals, thereby making its diagnosis difficult [4,5,6]. The ruminal acidosis caused by SCFAs is known as subacute ruminal acidosis (SARA). Notably, although the ruminal lactic acid may increase, it is not the cause of pH reduction in SARA [7].

As the clinical symptoms of SARA in the herd are more subtle, animals can suffer repeatedly from this “silent” condition, which will decrease their performance and reduce their weight gain [8,9,10]. In addition to this, SARA causes the reduction of several commensal cellulolytic rumen bacteria species [11].

In the long run, these repeated disease manifestations can cause inflammatory conditions such as laminitis and ruminitis. Vechiato et al. [12] studied slaughtered cattle from feedlots and found that 12% of inspected animals had inflammatory lesions in the rumen wall with the main cause of ruminitis being SARA. These lesions result in a decrease in nutrient absorption, thereby reducing the performance of the animals [13]. SARA is characterized by a ruminal pH between 5.2 and 5.8, for a minimum duration of 5 h [14]. In this pH range, Gram-negative bacteria die and release endotoxins (lipopolysaccharides (LPS)) from their cytoplasmic membranes, which results the increase of some acute-phase proteins such as serum amyloid A. Furthermore, an inflammatory condition results from damage to the ruminal papillae, which are responsible for nutrient absorption [15].

Inductions are controlled study methods that can aid in understanding the mechanism of SARA. It is believed that the SARA induction model with the use of CP, as described by Barrêto Júnior et al. [5], requires quantitative adjustments to correct for larger body weights (BWs). This is done to cause an adequate manifestation of SARA with the accumulation of SCFAs and some production of lactic acid, thereby increasing fluid osmolarity and causing changes in feeding behavior, rumination time, decubitus time, and overall resting duration. Additionally, the feeding behavior and restoration of normal feed intake after the occurrence of SCFA acidosis varies widely among individuals [10,16]. Here, we studied whether such recovery parameters were related to the amount of SCFA and lactate produced during SARA. Thus, we aimed to evaluate the degree of rumen fermentation by correlating it with organic acids and monitoring possible behavioral, clinical, and laboratory-related changes in Nelore heifers subjected to the experimental induction of SARA using the CP model.

## 2. Material and Methods

### 2.1. Animals, Adaptation, and Feeding

Thirteen 3-year-old Nelore heifers with an average weight of 544.21 ± 46.83 kg were used. During the adaptation phase and the experimental period, they remained in tie stalls.

Each heifer was fitted surgically with a ruminal cannula, and the animals received nonsteroidal anti-inflammatory drugs and antibiotics post-surgery. After recovery, the animals went through a period of adaptation to feed and environment conditions. Their basal diet was calculated at 2.5% of their live weight and comprised 75% of dry matter (DM) of coast-cross (*Cynodon dactylon*) hay and 25% of commercial concentrate (consisting of 80% corn meal and 20% soybean meal). The diet was offered twice a day. The animals had free access to water and a commercial mineral supplement. The CP used in the study for SARA induction had the following composition: 87.5% DM, 10.77% ash, 8.65% crude protein, 16.8% crude fiber, 21.25% neutral detergent fiber (NDF), 1.42% calcium, 0.18% phosphorus, and 0.11% magnesium.

### 2.2. Experimental Design

After the adaptation period, the animals were subjected to the SARA experimental induction model, through the sudden intraruminal administration of pelleted CP according to the induction protocol proposed by Barrêto Júnior et al. [5]. The SARA induction protocol using CP proposes the administration of CP directly into the rumen, at once, in amounts according to the animal body weight. The study from Barrêto Júnior et al. [5] indicates the amount of CP corresponding to 1.65% of the animal BW. This protocol was effective in inducing SARA in steers weighing 160 kg and led to the detection of ruminal pH values between 5.1 and 5.6 within a duration of 3 h or more. Therefore, the corresponding amount of CP recommended above was initially tested in a 450 kg pilot cow. Nine hours after induction, the ruminal pH presented values lower than 5.2, and by 18 h after induction, the pH value reached 4.74. This is typical of ruminal lactic acidosis; therefore, to obtain an adequate manifestation of SARA, lower CP doses were required. After performing tests on three other similar pilot cows, ruminal pH values of 5.2–5.8, which are characteristic of SARA, were obtained 3–9 h into the experiment for a minimum period of 5 h [14]. From the results obtained from these three cows, the following equation was achieved: Y (g) = BW^0.75^ × 54.7, where Y is the amount of CP in g; BW^0.75^ is the metabolic weight (used to correct different weights), and 54.7 is a corrective factor [16]. Thus, as an example, a heifer with 500 kg of BW would receive 5781.8 g of CP directly into the rumen for the induction of SARA.

### 2.3. Evaluation Time Points

**Figure 1:**
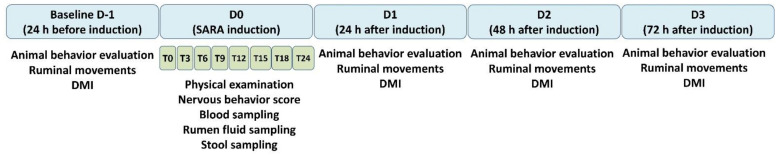
Experimental design, evaluated moments, and sampling procedures.

The animals were evaluated for a total period of five consecutive days: day zero (D0), which was the SARA induction day, and 4 days of behavioral evaluations, which comprised 1 day before (D-1) and 3 days after the induction (D1, D2, and D3) (Figure 1). On D0, the physical examination of the animals as well as rumen content, feces, urine, and blood sample collections were performed at the following time points: T0 (baseline moment before induction), T3, T6, T9, T12, T15, T18, and T24 (3, 6, 9, 12, 15, 18, and 24 h after induction, respectively). Meanwhile, during the physical examination, the following variables were measured: heart rate (HR), respiratory rate (RR), rectal temperature (T), and ruminal movement [17]. During D0, the animals were also evaluated for the degree of nervous behavior exhibited during SARA induction, according to Danscher et al. [18], with scores of 1, 2, 3, and 4 assigned to represent a dying animal, a very depressed animal, a slightly depressed animal, and an alert and responsive animal, respectively.

At D-1, D1, D2, and D3, the behavior of the animals was evaluated every 5 min for a 24 h period. The behaviors recorded were feed intake, rumination (in a standing or decubitus position), and resting in a standing or decubitus position [19]. After noting the findings of Danscher et al. [18], who discovered that SARA interfered with ruminal movement in the days following the induction, we decided to evaluate ruminal movement (always 9 h after the morning feeding) on D-1, D1, D2, and D3. During the experimental period, the animals remained in their respective pens with access to hay and water ad libitum.

### 2.4. Blood Sampling

Tubes containing EDTA and sodium fluoride were used for blood sampling. EDTA plasma was used for l-lactate and d-lactate, and plasma from sodium fluoride tubes were used for glucose analysis. The plasma l-lactate (levogyre) and glucose were determined using a commercial enzymatic kit (l-lactate-LAC and Randox Glucose GOD-PAP, respectively; Randox^®^, Crumlin, UK) in an automatic biochemical analyzer (Rx Daytona; Randox^®^). Further, a d-lactate (dextrogyre) enzyme assay (BioVision^®^, Milpitas, CA, USA) was used to measure the d-lactate plasma concentration.

### 2.5. Ruminal Fluid Sampling

Ruminal fluid samples were collected from three different rumen regions, namely, the cranial, medium, and caudal regions, using a plastic probe that was attached to a vacuum pump. Ruminal fluid was then immediately strained through sterile gauze, and ruminal pH and redox potential (E_h_) were analyzed immediately after sampling using a bench pH meter (DM-22; Digimed^®^, São Paulo, Brazil). Subsamples were frozen for additional analysis. The osmolarity (mOsm/L), d-lactates, l-lactates, and SCFA concentration were measured in all ruminal fluid samples.

To determine the SCFA concentration, 50 mL samples of ruminal fluid were centrifuged at 1000× *g* for 15 min, with a 1600 µL aliquot taken from the supernatant and added to 400 µL of formic acid p.a. (Dinâmica, São Paulo, Brazil). The solution was stored in microtubes to further analyze the SCFAs, the determination of which was carried out using gas chromatography [20]. In doing so, the propionic, acetic, butyric, isobutyric, valeric, and isovaleric acid values were determined. Meanwhile, the osmolarity of the ruminal fluid and serum was determined by freezing point depression on the Advanced Micro Osmometer 3300 (Advanced^®^, Norwood, MA, USA).

### 2.6. Feces Sampling

The pH of the stool samples was evaluated using a bench pH meter (DM-22; Digimed^®^, São Paulo, Brazil). The feces were assigned the following scores: 1, diarrheal; 2, pasty; 3, slightly firm; 4, hardened.

### 2.7. Determination of Ruminal Acidosis Caused by SCFAs

Ruminal acidosis caused by SCFAs was determined by identifying whether a ruminal pH in the range of 5.2–5.8 with a duration of up to 5 h was achieved [14]. The ruminal pH was measured using a bench pH meter and a rumen logger (Dascor^®^, Oceanside, CA, USA), which is a continuous pH measurement sensor calibrated with the M5-v760 software (Dascor^®^) used to measure the ruminal pH every 5 min. The rumen logger was placed in the ventral bag so that the sensor remained in contact with the ruminal fluid throughout the experimental period.

### 2.8. Statistical and Validation Analysis

The statistical analysis was performed using the Minitab Release 19 (Minitab^®^ Inc, State College, PA, USA) statistical program. The data were initially evaluated for their distribution using the Kolmogorov–Smirnov test. Tukey’s multiple comparison test was used for data exhibiting normal distribution, to evaluate the difference between the experimental time points; while the Mann–Whitney test was used to analyze the data with non-normal distribution. Owing to the presence of several null results, the ruminal movement needed to be transformed by the square root (√*x* + 1).

The correlation between some of the variables was determined using Spearman’s correlation coefficient (*r*) of the coefficient of determination (*r*^2^) with the level of significance set at 5%. Validation and the results of calibration from the laboratory analysis used in this study are shown in the Supplementary Materials (Table S1). 

## 3. Results

### 3.1. Ruminal Variables

The complete ruminal profile is presented in Table 1. The animals subjected to SARA had a higher pH at T0, which then decreased and reached its lowest values between T6 and T9. The time point at which the animals had ruminal pH values within the SARA threshold (i.e., pH = 5.2–5.8) was at 547 ± 215 min, with a range of 305–940 min. Moreover, the minimum pH value detected was 5.38 ± 01.16, and the average pH was 5.62 ± 0.10.

The average acetic acid levels reached their maximum value at T3, followed by the other time points examined in the experiment, which were all higher than the baseline time. However, the ruminal concentrations of propionic acid were lower at baseline than at other times, which were similar to each other. Furthermore, while the butyric acid content varied slightly, in general, it was higher in all the time points than at the baseline time point, with values at T12 and T15 being higher than those at T3. Additionally, there was no difference in the isobutyric acid concentration during the experiment, and the total SCFA content was found to be the lowest at the baseline.

In the study of the relationship between the SCFAs, a high propionic acid concentration led to high butyric acid (*r* = 0.643; *p* < 0.0001) and acetic acid (*r* = 0.46; *p* < 0.0001) concentrations as well. Moreover, although there was a positive and significant relationship (*p* = 0.02) between the acetic and butyric acid concentrations, the correlation coefficient was considered to be low (*r* = 0.261).

The acetate/propionate ratio was lower from T9 to T18 than from baseline to T3. The l-lactate levels peaked at T6 and T9, reflecting levels that were higher than at other time points. Meanwhile, the d-lactate levels were lower at baseline than at T3, T6, and T9, and the average ruminal glucose content was the highest at T3.

The E_h_ level was higher at T6 than at all other time points, except at T9, when it was greater than that at baseline, T15, T18, and T24. Moreover, the ruminal pH showed a high negative correlation with E_h_ (*r* = −0.97; *p* < 0.0001).

The osmolarity was higher at T3 and T6 than at all other time points, with the third highest time being T9, while the ruminal osmolarity was highly correlated with l-lactate (*r* = 0.67; *p* < 0.0001), glucose (*r* = 0.63; *p* < 0.0001), and acetic acid (*r* = 0.41; *p* < 0.0001) concentrations. The correlation between pairs of ruminal variables found in the present study is presented in detail in Table 2.

### 3.2. Animal Behavior

The variables used for behavior evaluation are presented in Table 3. The dry matter intake (DMI) in the first 2 days after SARA induction (D1 and D2) was directly correlated with the minimum ruminal pH during SARA induction (D1: *r* = 0.63, *p* = 0.05; D2: *r* = 0.65, *p* = 0.042) but was inversely correlated with the duration of SARA (D1: *r* = −0.473, *p* = 0.033; D2: *r* = −0.514, *p* = 0.035). The lower the mean pH was during SARA, the greater was the depression in the animals’ appetite on D1 (*r* = −0.823; *p* = 0.009) and D2 (*r* = 0.63; *p* = 0.012). The lower the DMI was on D1, the lower it was on D2 (*r* = 0.823; *p* < 0.0001) as well. Additionally, the higher the individual average ruminal osmolarity on D-1 and D1, the lower was the DMI (*r* = −0.739; *p* < 0.0001).

The time it took to consume 1 kg of DM was longer on D1 and D2 than on D−1 and D3. Furthermore, the time spent ruminating was higher (*p* < 0.0001) on D-1, followed by D3, during which it was higher than on D1 and D2. Moreover, there was a positive relationship between the ruminal pH on D-1 and D0 and the rumination time (minutes) during these periods (*r* = 0.899; *p* < 0.0001).

The total resting time was greater on D1 than on D-1 and D3 (*p* = 0.002). There was no difference in the resting time duration between the animals in the standing and the lying position (*p* = 0.679). However, the resting time in decubitus was more prolonged on D1 than on D-1 and D3; while on D2 it was only longer than that measured on D-1 (*p* = 0.001). The lower the minimum ruminal pH during SARA, the longer the cattle remained in decubitus on D1 (*r* = −0.683; *p* = 0.03). Additionally, it was found that the greater the appetite, the shorter was the time that cattle remained in decubitus (*r* = −0.716; *p* < 0.0001).

### 3.3. Clinical Variables

The Table 4 presents the results from clinal variables during the study. During the induction of SARA (D0), there was a punctual heart rate increase at T9 and a respiratory rate increase at T6 and T9, in relation to T0. The rectal temperature was higher at T6 and T9 than at all other time points, except for T12, when the rectal temperature was identical to that at the other time points, but higher than at T0. When the relationship between the ruminal pH on D0 and the rectal temperature was evaluated, a significant negative correlation was found (*r* = −0.714; *p* < 0.0001). The ruminal movement at T0 was higher than at the other time points during induction. The greater the DMI during the experiment, the greater was the number of ruminal movements (*r* = 0.860; *p* = 0.0001), and there were no differences in the fecal pH and score.

During the assessment of the behavioral status of the cattle on the first day of acidosis, there were different nervous behavior changes of short-term duration. The relationship between these different nervous behavior changes and some variables were, therefore, examined. The lower the minimum ruminal pH was, the lower was the degree of depression (*r* = 0.80; *p* = 0.002). Additionally, we found that the longer the decubitus time, the lower was the degree of depression (*r* = −0.785; *p* = 0.002), and the higher the maximum ruminal d-lactate content, the more depressed the animal was (*r* = −0.621; *p* = 0.05).

### 3.4. Blood Variables

Blood glucose levels were higher at T18 than at baseline but remained within the physiological reference interval [21]. There were no differences in blood l-lactate concentrations during the induction of SARA. However, there was a positive relationship between the organic acid sum, and ruminal glucose and blood glucose contents (*r* = 0.436; *p* < 0.0001).

## 4. Discussion

### 4.1. Adequacy of the Induction Model and Ruminal Variables

It was necessary to remodel the protocol proposed by Barrêto Júnior et al. [5] for SARA induction because the weight of the animals selected for this experiment was higher than that of the animals used by the aforementioned authors. The adaptation was based on the findings of Ortolani [22], who tested the model of ruminal lactic acidosis (RLA) induction with sucrose and observed that heavier animals were more predisposed to RLA. Based on this finding, we performed tests to correct the amount of sucrose initially used for metabolic weight to allow for a more uniform induction, regardless of BW. The first animal tested in this study, which was administered the original CP dose proposed [5], suffered from RLA. Therefore, the CP dose was recalculated to obtain a ruminal pH between 5.2 and 5.8 for at least 5 h, which was in accordance with the characteristics of SARA [14].

The ruminal pH results obtained from this study included a minimum pH of 5.38 ± 0.16, an average pH of 5.62 ± 0.1, and a case of SARA lasting 547 ± 215 min. Previous studies used wheat and barley in SARA inductions and obtained a minimum pH of 5.1–5.6, an average pH of 5.4–5.8, and an average SARA duration that was less than 490 min [18,23,24,25].

The total SCFA generation had a maximum value of 118.4 mmol/L, which was similar to that described in other studies [18,25]. However, concentrations as high as 130.5 and 137.2 mmol/L, which generated a pH lower than 5.1, have also been described [23,24].

The fermentation of CP, mainly pectin, resulted in an increase in the levels of three SCFAs, particularly acetic acid, while also producing lactic acid. In vitro experiments have shown that the water-soluble extract of CP can undergo great ruminal fermentation within 12 h, thereby decreasing the pH of the medium to 5.13 and resulting in high l-lactic and lower d-lactic acid production [6]. In this study, the excess CP decreased the ruminal pH and resulted in the significant production of lactic acid. Moreover, in some instances, the l-lactic and d-lactic acids reached values higher than 50 and 13 mmol/L, respectively, 6 h into the experiment when the pH was lower. Further, at pH values below 5.5, there is a decrease in the activity of lactic acid bacteria with the growth of *Lactobacillus* and some activity of *Streptococcus bovis*, which produces l-lactic acid and, to a lesser degree, d-lactic acid [26,27].

However, the pH reduction that took place 6 h into the experiment did not continue. This probably occurred because there was a decrease in the levels of energy substrate that was measured indirectly by ruminal glucose, which returned to baseline values 6 h into the experiment, thus, not providing an energy source for the generation of lactic acid. Additionally, the discontinuation of the pH reduction could be attributed to the absorption of SCFAs, which led to the removal of significant amounts of various acids from the rumen.

The ruminal pH reduction was not only caused by the total SCFA concentration, or by its main acids, but also by l-lactic acid. The coefficient of determination (*r*^2^) identified that 31%, 29%, and 25% of the pH drop was caused by the production of lactic, propionic, and acetic acids, respectively. Although the lactic acid concentration was much lower than that of the main SCFA, the pK_a_ of lactic acid is approximately 10 times higher than that of the propionic and acetic acids [28]; although the acetic acid production was higher than that of the propionic acid, the latter had a slightly more significant role than acetic acid in decreasing rumen pH, as the propionic acid pK_a_ is 1.6 times higher than the acetic acid pK_a_ [29].

The maximum propionic acid value and the lowest acetic/propionic ratio were similar to those achieved in previous research on SARA [18,25]. Notably, there was a relationship observed between the increase in the production of the propionic and butyric acids, and the propionic and acetic acids. A diet rich in CP increases the propionic and butyric acid levels [30]. Among the SCFA analyzed numerically, the presence of valeric acid, which had a higher concentration at T9 and T12 in relation to T0, is noteworthy. This increase occurs owing to the activity of lactic acid bacteria that transform lactic acid into valeric acid to a small extent, and this may have had some diagnostic significance [29].

The E_h_ was influenced by the ruminal pH and became positive during the course of SARA. Huang et al. (2018) also reported that the lower the ruminal pH, the higher the E_h_, as this value increased by 60% when the pH decreased from 6.8 to 5.8. Additionally, Marden et al. (2013) studied the E_h_ of cows with SARA and verified an increase of the minimum ruminal pH of up to 5.8. Acidosis decreases Gram-negative bacteria and increases Gram-positive bacteria, especially *Streptococcus bovis* and *Lactobacillus* sp., which are capable of growing in a more aerobic environment. With the pH decrease, the death or reduced activity of Gram-negative bacteria occurs, and both occurrences increase the O_2_ tension in the medium, thereby increasing the E_h_; this is especially so with the reduced activity of Gram-negative bacteria [31].

Osmolarity was high between T3 and T6 when acidosis was at its peak. The correlation between osmolarity and the other ruminal variables helps to explain the increase observed in this concentration in the rumen fluid. Further, the correlation coefficient with l-lactic acid, glucose, and acetic acid stood out. Coincidentally, osmolarity was numerically higher 3 h into the experiment when the ruminal glucose content had an isolated peak incidence.

### 4.2. Behavioral and Clinical Variables

SARA caused a decrease in feed intake as it led to a substantial appetite reduction on D1 (66.34%) and D2 (48%). Nevertheless, the appetite levels returned to normal on D3. This finding has also been described by some authors [9,29]. Brown et al. [32] induced acidosis with the ruminal administration comprised 50% of energy-rich grains and observed the restoration of appetite on the third day. However, the decrease in food intake was not as marked as it was in this study.

Low pH levels, especially less than 5.5, provoked an increase in the ruminal lactate levels and in osmotic pressure as well as a reduction in the frequency and amplitude of ruminal contractions. This condition, combined with the inflammation of the ruminal epithelium, may have been the cause of the decreased appetite [3,12,29]. The first important result of this study was that the degree of hyporexia on the first day determined the appetite on the second day of SARA, a fact not previously described. That is, the greater the physical and chemical aggression of SARA, the greater the interference on the following day. It is important to note that appetite is one of the most sensitive and changeable variables in the face of injuries caused by diseases, pain, stress, and other inflammatory responses [33].

The current study compared data on food intake from three ruminal pH behavior evaluations during SARA; the influence of minimum pH, the duration of acidic pH, and the average pH during acidosis. It was found that the minimum pH was less influential than the average pH during acidosis on the DMI. In addition, the duration of acidosis was found to have a minimal influence on feed intake. The minimum pH was expected to be the main factor in reducing appetite; however, it was less influential than the average pH. On D2, both the minimum and the average pH had a similar influence on food intake, with the duration of acidosis continuing to have a low impact on food consumption.

It was found that the lower the ruminal movement, the lower the level of food intake. Desnoyers et al. [34] stated that the low frequency and amplitude of the ruminal movement generated by acidosis potentiates the reduction of DM consumption in cattle. This would increase the rumen volume, consequently decreasing the digesta passage rate, which would then lead to a reduction in food intake [35]. The same phenomenon occurs in cattle that receive a diet rich in acid detergent fiber, which is difficult for the rumen to digest, thereby increasing the retention time in the rumen and decreasing the intake of DM [36].

The increase in ruminal osmolarity could also interfere with food intake [29,34]. This is evidenced by the fact that as the average osmolarity increased during SARA on D1, the food intake saw a decrease. In the musculature of the cranial sac of the rumen and the reticulum, some baroreceptors can detect osmolarity up to 500 mOsm/L. Notably, osmolarity levels higher than 300 mOsm/L have a negative correlation with food intake [37]. Another notable result of this study was the time it took to ingest 1 kg of DM. On D1 and D2, the ingestion of an equal amount of food was much slower than in the basal period and on D3. During the behavioral observations, cattle that were the most affected by SARA were the most lethargic and depressed and took longer to eat.

The rumination time was shorter on D1 and D2 than on D-1 and D3. Similar findings were obtained in cows on the first but not on the second day of acidosis, with a reduction in rumination duration by 18% [38]. In contrast, the corresponding reduction in this study was 58%. Rumination time had a positive correlation with food intake and the average ruminal pH (but this relationship was negatively correlated with the time it took to ingest 1 kg of DM).

The total time the cows spent resting increased on D1 compared to D-1 and D3, mainly owing to the longer decubitus duration; the same increase was true for D2 when compared to D-1. SARA had no influence on the time that the animals remained standing. Acidotic animals tend to stand up more and lie down less than they do before acidosis [38]. However, this is contrary to the findings of this study.

The most depressed cattle were those that spent the most time in decubitus. The lower the minimum ruminal pH on D1, the longer the animal was in decubitus during this period; consequently, the greater the depression, the longer the animal was in decubitus. Furthermore, the more severe the case of acidosis, the greater the degree of depression [33]. The heart rate increased slightly at T9 compared to baseline, and similar to the cardiac variable, the respiratory rate had a punctual and discreet increase at T6 and T9. However, this result did not have great diagnostic value. The same occurred with the rectal temperature, which, in some cases (*n* = 5) reached values higher than 39.5 °C, which is considered normal for cattle. The rectal temperature was negatively correlated with the ruminal pH, thereby indicating that the temperature increase generated by high rumen fermentation somehow interfered with systemic temperature. This finding was previously described in another study [39] in which the authors induced RLA in sheep and found that at the peak of rumen fermentation (pH 4.8–5.5), the rumen temperature rose to 40.5 °C, thereby increasing the systemic temperature.

The rumen movement was always higher at baseline in relation to all the other time points on D1. Further, the lowest ruminal movement was detected on D1, while on D2 the ruminal movement frequencies were lower than baseline, returning to normal on D3. Taurine cattle maintain some ruminal motility regardless of the degree of acidosis, while Zebu cattle have atony, which favors dehydration by preventing the absorption of lactic acid but increases ruminal osmolarity [16].

Neither the fecal pH nor the fecal score changed during SARA, which is different than that found in previous studies [18] that detected a slight drop in fecal pH and the presence of softer stools in some of the animals. The fecal pH is more acidic only in cases where the presence of soluble sugars is high, because of the automatic passage (by-pass) through the rumen, which is not always the case with SARA [9]. 

SARA caused an increase in osmolarity, which generated a decrease in rumen movement on the first day of the experiment. An increase in osmolarity higher than 350 mOsm/L negatively affects ruminal motility, which includes the time spent ruminating [37].

Blood glucose was punctually higher at T18 than at baseline. In ruminants, approximately 60% of the gluconeogenic substrate is produced from ruminal propionate; the rest is produced by certain gluconeogenic amino acids, such as l-lactate and glycerol, and some by-pass glucose that enters the intestines. In the present experiment, there was an increase in glucose in the rumen at T3; however, it is certain that this glucose was transformed into other SCFAs and lactic acid, which made its absorption by this organ impossible.

The blood l-lactate results did not change over time, probably owing to the rapid systemic metabolism of this compound being oxidized or transformed into glucose [40].

## 5. Conclusions

The experimental SARA induction using CP was characterized by a reduction in the ruminal pH owing to the accumulation of SCFAs, especially acetic acid, with a limited amount of lactic acid produced. SARA caused a reduction in food consumption and rumination duration, as well as an increase in the time spent in decubitus during the first 2 days of the condition. These results were influenced by the ruminal pH, ruminal movement, and osmolarity. Furthermore, SARA caused different levels of depression (apathy/lethargy), which became more pronounced when the ruminal lactic acid concentration was higher. The heart and respiratory rates, pH and fecal scores, blood glucose concentrations, and l-lactate levels were found to be unsuitable markers for the clinical diagnosis of SARA.

## Figures and Tables

**Table 1:** Ruminal variables of cattle subjected to subacute ruminal acidosis (SARA) induction with the use of citrus pulp.

Variables	Evaluation Moments							
	T0	T3	T6	T9	T12	T15	T18	T24
pH	6.93 ± 0.1 ^a^	5.84 ± 0.1 ^b^	5.5 ± 0.1 ^b^	5.59 ± 0.2 ^b^	5.82 ± 0.2 ^b^	6.02 ± 0.2 ^b^	6.16 ± 0.2 ^b^	6.36 ± 0.3 ^b^
Aa (mmol/L)	55.1 ± 6.2 ^c^	83.8 ± 7.2 ^a^	69.4 ± 4.7 ^b^	70.3 ± 8.4 ^b^	66.2 ± 7.0 ^b^	71.5 ± 6.6 ^b^	73.4 ± 9.0 ^b^	71.9 ± 8.3 ^b^
Pa (mmol/L)	12.0 ± 2.2 ^b^	22.4 ± 3.7 ^a^	21.1 ± 3.6 ^a^	24.7 ± 4.7 ^a^	25.6 ± 3.7 ^a^	29.0 ± 5.3 ^a^	26.3 ± 5.5 ^a^	24.2 ± 6.5 ^a^
Ba (mmol/L)	5.7 ± 0.9 ^d^	9.9 ± 1.2 ^c^	11.1 ± 1.6 ^bc^	13.5 ± 2.4 ^bc^	13.9 ± 3.7 ^ab^	14.0 ± 3.2 ^ab^	13.3 ± 3.8 ^bc^	10.3 ± 3.8 ^bc^
SCFA (mmol/L)	75.1 ± 9 ^b^	117.3 ± 13 ^a^	103.7 ± 9 ^a^	111.2 ± 14 ^a^	101.8 ± 21 ^a^	117.2 ± 7 ^a^	118.4 ± 9 ^a^	109.6 ± 14 ^a^
Aa/Pa ratio	4.7 ± 0.8 ^a^	3.7 ± 0.7 ^b^	3.3 ± 0.4 ^bc^	2.9 ± 0.3 ^c^	2.6 ± 0.3 ^c^	2.6 ± 0.8 ^c^	2.9 ± 0.7 ^c^	3.1 ± 0.8 ^c^
GLU (mmol/L)	0.74 ± 0.06 ^b^	7.07 ± 5.52 ^a^	0.80 ± 0.07 ^b^	0.81 ± 0.11 ^b^	0.79 ± 0.09 ^b^	0.81 ± 0.12 ^b^	0.77 ± 0.09 ^b^	0.73 ± 0.08 ^b^
l-Lac (mmol/L)	0.12 ± 0.02 ^c^	1.17 ± 0.5 ^b^	7.17 ± 1.3 ^a^	6.16 ± 0.9 ^a^	3.07 ± 0.5 ^b^	0.49 ± 0.01 ^bc^	0.09 ± 0.01 ^c^	0.05 ± 0.01 ^c^
d-Lac (mmol/L)	0.07 ± 0.01 ^b^	0.42 ± 0.02 ^a^	0.56 ± 0.02 ^a^	0.47 ± 0.02 ^a^	-	-	-	-
E_h_ (mV)	4.0 ± 7.3 ^e^	65.2 ± 20.8 ^c^	95.9 ± 15.6 ^a^	89.5 ± 16.1 ^ab^	75.7 ± 13.1 ^bc^	64.8 ± 14.3 ^cd^	56.3 ± 13.1 ^cd^	47.5 ± 19.9 ^d^
OSM (mOsm/L)	276 ± 30.5 ^c^	405 ± 45.2 ^a^	377 ± 23.9 ^a^	335 ± 27.5 ^ab^	304 ± 18.3 ^bc^	290 ± 20.1 ^c^	284 ± 14.8 ^c^	279 ± 7.2 ^c^

Different lowercase letters on the line denote differences between the assessment times (*p* < 0.05). T0 to T24 indicate the time in hours after the SARA induction. Aa: acetic acid; Pa: propionic acid; Ba: butyric acid; SCFA: short-chain fatty acid; GLU: glucose; l-Lac: l-Lactate; d-Lac: d-Lactate; OSM: osmolarity.

**Table 2:** Correlation between pairs of ruminal variables in beef heifers submitted to experimental induction of subacute ruminal acidosis.

Variables	E_h_	Osmolarity	Glucose	l-Lactate	PROP	BUTYR	ACETIC	Total SCFAs
pH	−0.97 *p* < 0.0001	−0.64 *p* < 0.0001	−0.08 *p* = 0.50	−0.56 *p* < 0.0001	−0.54 *p* < 0.0001	0.02 *p* = 0.89	−0.37 *p* < 0.05	−0.52 *p* < 0.0001
E_h_		0.54 *p* < 0.0001	0.06 *p* = 0.57	0.59 *p* < 0.0001	0.59 *p* < 0.0001	−0.01 *p* = 0.97	0.34 *p* = 0.12	0.55 *p* > 0.001
Osmolarity			0.63 *p* < 0.0001	0.67 *p* < 0.0001	0.08 *p* = 0.50	0.01 *p* = 0.96	0.41 *p* <0.0001	0.26 *p* = 0.20
Glucose				0.25 *p* = 0.03	0.02 *p* = 0.89	−0.12 *p* = 0.30	0.43 *p* < 0.0001	0.24 *p* = 0.03
l-Lactate					−0.02 *p* = 0.88	−0.01 *p* = 0.98	0.02 *p* = 0.86	−0.05 *p* = 0.67
PROP						0.57 *p* < 0.0001	0.46 *p* < 0.0001	0.81 *p* < 0.0001
BUTYR							0.26 *p* = 0.02	0.64 *p* < 0.001
ACETIC								0.83 *p* < 0.0001

E_h_: redox potential; PROP: propionic acid; BUTYR: butyric acid; ACETIC: acetic acid; SCFAs: short-chain fatty acids.

**Table 3:** Behavioral variables of cattle subjected to subacute ruminal acidosis (SARA) induction with the use of citrus pulp.

Variables	Evaluation Moments			
	D-1	D1	D2	D3
Dry matter intake (kg)	10 ± 1.23 ^a^	3.4 ± 1.9 ^b^	5.2 ± 2.9 ^b^	8.8 ± 1.8 ^a^
Time for the intake 1 kg of DM (min)	32 ± 4 ^a^	94 ± 23 ^a^	90 ± 31 ^a^	28.5 ± 3.4 ^b^
Rumination time (min)	450 ± 68 ^a^	187 ± 63 ^c^	231 ± 87 ^c^	356 ± 67 ^b^
Resting at standing position (min)	220 ± 76	251 ± 109	250 ± 12	229 ± 14
Resting at decubitus (min)	380 ± 60 ^c^	528 ± 127 ^a^	481 ± 34 ^ab^	418 ± 55 ^bc^
Resting time (min)	600 ± 101 ^c^	779 ± 178 ^ab^	731 ± 46 ^bc^	628 ± 71 ^c^
Ruminal movements (mov/3 min)	3.56 ± 0.4 ^a^	1.56 ± 0.73 ^c^	2.77 ± 0.76 ^b^	3.19 ± 0.37 ^ab^

Different lowercase letters on the line indicate differences between assessment times (*p* < 0.05). D = day.

**Table 4:** Mean values and standard deviation of the heart rate (HR), respiratory rate (RR), temperature (T), ruminal movements (RM), and blood glucose of cattle subjected to subacute ruminal acidosis (SARA) induction with the use of citrus pulp.

Variables	Evaluation Moments							
	T0	T3	T6	T9	T12	T15	T18	T24
HR (bpm)	78 ± 6 ^b^	90 ± 14 ^ab^	89 ± 14 ^ab^	96 ± 16 ^a^	89 ± 12 ^ab^	86 ± 14 ^ab^	81 ± 9 ^ab^	80 ± 7 ^a^
RR (mpm)	22 ± 4 ^a^	31 ± 9 ^ab^	33 ± 9 ^a^	33 ± 9 ^a^	29 ± 6 ^ab^	26 ± 7 ^ab^	24 ± 5 ^ab^	25 ± 4 ^ab^
T (°C)	38.2 ± 0.3 ^c^	38.6 ± 0.4 ^bc^	39.4 ± 0.3 ^a^	39.2 ± 0.4 ^a^	39.0 ± 0.4 ^ab^	38.7 ± 0.4 ^bc^	38.4 ± 0.3 ^bc^	38.3 ± 0.3 ^bc^
RM (mov/3 min)	3.6 ± 0.4 ^a^	2.0 ± 0.7 ^b^	1.6 ± 0.7 ^b^	2.2 ± 0.9 ^b^	2.1 ± 0.8 ^b^	2.2 ± 0.9 ^b^	2.27 ± 0.4 ^b^	2.13 ± 0.8 ^b^
GLU (mmol/L)	4.3 ± 0.2 ^c^	4.9 ± 0.7 ^bc^	5.1 ± 0.5 ^bc^	4.7 ± 0.6 ^bc^	4.9 ± 0.5 ^bc^	5.0 ± 0.4 ^bc^	5.1 ± 0.6 ^ab^	4.7 ± 0.2 ^bc^

Different lowercase letters on the line indicate a difference between assessment times (*p* < 0.05). GLU: glucose.

## Data Availability

The raw data related to this study are available on request from the corresponding author.

## Supplementary Materials

The following are available online at https://www.mdpi.com/2076-2615/11/1/21/s1, Table S1: Analytical controls and calibration used in this study.
