# Supplementary material for: Subacute Ruminal Acidosis in Zebu Cattle: Clinical and Behavioral Aspects
Source: Animals (Basel). 2020 Dec 24;11(1):21. doi: 10.3390/ani11010021 (PMC7824239; doi:10.3390/ani11010021)
Supplement: Supplementary file 1 [file animals-11-00021-s001.pdf]

Title-**Subacute Ruminal Acidosis in Zebu Cattle: Clinical and Behavioral Aspects**

Supplementary Material S1—Analytical Controls and Calibration

Table S1. Analytical controls and calibration used in this study.

Variable	Calibration/Validation
L-Lactate	Biochemical kit (Randox®, Crumlin, UK). Calibrator C2 REF: CAL2350, Randox (1.776 mmol/L); Quality control normal N2 REF: HN1530, Randox (1.443–2.054 mmol/L), recovery >98%; Quality control pathological N3 REF: HE1532, Randox (1.776–4.218 mmol/L), recovery >97%.
D-Lactate	D-Lactate Colorimetric Assay Kit REF: K667 (BioVision Inc., Milpitas, CA, USA). D-Lactate calibration standard (100 mmol). $r^2 = 0.98$. Calibration curve from 0.01 to 10 mmol.
Glucose	Randox Glucose GOD-PAP assay kit (Randox®). Calibrator C2 REF: CAL2350, Randox: 5.42 mmol/L. Quality control normal N2 REF: HN1530, Randox: 4.78–6.45 mmol/L. Quality control pathological N3 REF: HE1532, Randox: 10.89–14.55 mmol/L, recovery >98%.
Osmolarity	Internal precheck calibration. Reference solution with 290 mOsm (Ref.: MA029). Acceptable variation: ± 2 mOsm. Quality control low: 50 mOsm Ref: 3MA005. Quality control high: 850 mOsm, Ref: 3MA085. Advanced®, Norwood, MA, USA.
pH	Standard calibration in each day using 4.01 pH, REF: DM-S1B and 7.00 pH, REF: DM-S1D. Digimed, São Paulo, Brazil.
SCFA	Calibration with reference standards. Acetic acid analytical standard, GC assay ≥ 99.8%, Sigma-Aldrich, San Luis, Mi, USA, REF: 71251. Propionic acid analytical standard, GC assay ≥ 99.5%, Sigma-Aldrich, REF: 94425. Butyric acid analytical standard, GC assay ≥ 99.5%, Sigma-Aldrich, REF: 19215.

SCFA: Short-chain Fatty Acid.
